# Factors influencing depression in community-dwelling elderly patients with osteoarthritis of the knee in China: a cross-sectional study

**DOI:** 10.1186/s12877-022-03117-0

**Published:** 2022-05-25

**Authors:** Xiaoyan Zheng, Yaya Wang, Xiaoyan Jin, Hongjie Huang, Hongbo Chen, Yan Wang, Shaomei Shang

**Affiliations:** 1 The Open University of China, 75 Fuxing Road, Haidian District, Beijing, 100039 China; 2 School of Nursing, Peking University, 38 Xueyuan Road, Haidian District, Beijing, 100191 China; 3 Institute of Sports Medicine, Peking University Third Hospital, 49 Huayuan North Road, Haidian District, Beijing, 100191 China

**Keywords:** Depression, Elderly, Osteoarthritis of the knee, Community, Influencing factors

## Abstract

**Background:**

Knee osteoarthritis (OA) and depression are both major health issues influencing the quality of elderly life. The aim of the present study was to explore the prevalence of depression and the factors influencing depression in community-dwelling elderly patients with OA of the knee in China.

**Methods:**

We conducted a cross-sectional descriptive study. The study included 214 participants aged 60 and older diagnosed with OA of the knee. The depression of the elderly was measured by using the Geriatric Depression Scale (GDS). Participants were asked to complete a demographic questionnaire, the GDS, the Western Ontario and McMaster Universities Osteoarthritis Index (WOMAC), the society dimension of Arthritis Impact Measurement Scales 2 (AIMS2). In addition, the participants performed a timed up and go test (TUG) and the stair-climb test (SCT).

**Results:**

The average age of the participants was 69.2 ± 7.63 years old, their body mass index (BMI) was 25.2 ± 3.85, and their disease duration was 5.9 ± 7.72 years. The mean total score of the GDS was 4.43 ± 2.89, and the GDS scores correlated positively with pain (*r* = 0.45, *P* < 0.001), stiffness (*r* = 0.40, *P* < 0.001), physical function (*r* = 0.52, *P* < 0.001),TUG (*r* = 0.35, *P* < 0.001), and SCT (*r* = 0.47, *P* < 0.001) and negatively with social support (*r* = − 0.35, *P* < 0.001).Analysis using multiple regression demonstrated that physical function, social support, and SCT explained 36.8% of the variance in depression.

**Conclusions:**

Our findings suggested that physical function, social support, and lower extremity strength were predictors of depressive symptoms in community-dwelling elderly people with OA of the knee. Focusing on this elderly group with increasing functional exercise, positive social interaction and support, and lower limb muscle strength training should help in the prevention of depression.

**Supplementary Information:**

The online version contains supplementary material available at 10.1186/s12877-022-03117-0.

## Background

Osteoarthritis (OA) is a chronic, degenerative, musculoskeletal disease with global prevalence of 20% of women and 10% of men over 60 years of age [1]. Most patients with OA of the knee will develop progressive functional limitation and physical disability with age [2]. The prevalence of symptomatic OA of the knee among elderly Chinese people was reported as 10.3% (women) and 5.7% (men) (adjusted odds ratio (OR) 1.88) [3]. The disabling symptoms of knee OA in older patients, such as chronic pain, joint stiffness, and physical dysfunction, potential to cause psychological changes in patients, leading to depression [4]. Indeed, a recent study showed that long-term OA was one of risk factors for incident depression in community-dwelling adults [5].

It was estimated that among communities of elderly adults, significant symptoms of depression could be detected in 8 to 16% of them [6]. Prior studies have shown that depression of individuals with OA can exacerbate pain, reduce function, and have an additive adverse impact on health-related quality of life, disability, and response to treatment [7–9]. In addition, depression could impose a significant disease burden among individuals with arthritis, their families, and society as a whole [10]. Thus, in elderly patients with knee OA and depression, appropriate screening, assessment, and treatment is required [6].

Depression in community dwelling older patients was affected by a multiple of factors, including age, sex, pain, impaired mobility, low education, comorbidity, and social support [11–13]. In addition, previous studies of elderly patients with OA of the knee had shown a significant association between depression and obesity, a longer walking time, and a slower walking speed [14–16]. Increasing our understanding of the occurrence of depression and factors that influence it among patients with OA of the knee will help to develop interventions to alleviate depression and improve these patients’ quality of life. Therefore, the aim of the present study was to determine the prevalence of depression and identify the factors influencing depression among older community-dwelling patients with OA of the knee in China.

## Methods

### The design of the study and recruitment of the study population

This study used a cross-sectional, descriptive, correlational design. Participants were recruited from three major communities in Beijing using convenience sampling. In the three communities, elderly patients with OA of the knee were asked to join the study. The inclusion criteria were: (a) age 60 years and older and (b) diagnosed with OA of the knee [17, 18]. The exclusion criteria were: (a) under antidepressant treatment, (b) unable to take part in the tests due to symptoms of severe disability, visual impairment, or nerve dysfunction, or (c) combined with a severe disease, such as Parkinson disease, cancer, or stroke. Power analysis was conducted to determine the sample size using PASS2021 software (NCSS LLC, Kaysville, UT, USA) for multiple regression. We tested 22 predictive variables and the regression model (R [2]) was set at 0.2 to explain the amount of variation. To achieve a power of 0.8 (alpha = 0.05), 91 participants (minimum) were required.

### Measures

All the participants completed questionnaires, including demographic information, the Short-Form Geriatric Depression Scale (GDS), the Western Ontario and McMaster Universities Osteoarthritis Index (WOMAC), and the society dimension of Short-Form Arthritis Impact Measurement Scales 2 (AIMS2). In addition, the participants performed a timed up and go test (TUG) and the stair-climb test (SCT).

This study developed a demographic sheet to collect personal information, including age, sex, body mass index (BMI), marital status, ethnicity, residence, educational level, average monthly household income, medical expense, comorbidities, duration of the disease, number of diseased knees, a number of falls in the past year, use of walking aids, and consumption of pain relievers. We also investigated the level of occupational activity before retirement, such as light physical activity (teachers, civil servants, etc.) and heavy physical activity (workers, farmers, truck driver, etc.).

The depression of the elderly was measured by using the Chinese version of the GDS [19]. This scale comprises 15 self-reported measures about the patients’ feelings regarding their daily lives [20]. The answers were labeled as ‘yes’ (1) or ‘no’ (0) [6]. ‘Yes’ represented a positive score for depression in 10 of the questions; whereas, ‘no’ represented a positive score for depression in the other five questions, e.g., ‘Are you basically satisfied with your life?’ ‘Do you think it is wonderful to be alive now?’. The total score could range from 0 to 15, in which more depressive symptoms were indicated by higher scores [21]. A score ≥ 5 defined mild depression, and a score ≥ 11 defined moderate/severe depression [14]. Sensitivity and specificity of the GDS have been assessed in many studies and have been proven to have good validity and reliability [22–24]. When used in community-dwelling older adults, the Chinese eversion of the GDS had a split-half reliability of 0.84 and a Cronbach’s alpha score of 0.90 [19].

Disease extent was assessed using the WOMAC index [25], which comprises a self-assessment questionnaire with 24 items on three subscales: pain (five items, score range 0–20), stiffness (two items, score range 0–8), and physical function (17 items, score range 0–68). Higher scores indicated increased pain and stiffness, and worse physical function. The Chinese version of WOMAC has strong internal consistency (Cronbach’s alpha = 0.84–0.96) and acceptable test-retest reliability, as indicated by the intraclass correlation coefficients (ICC = 0.76–0.85) for all domains [26]. For the WOMAC index, the present study achieved a Cronbach’s alpha score of 0.95, and a split-half coefficient of 0.80.

To measure the respondents’ social support, the society dimension of AIMS2 was used. AIMS2 measures arthritis patients’ quality of life using a self-assessment scale [27], comprising 26 items measured on a 5-point Likert scale. The society dimension contains four items, which are ‘Often get together with friends or relatives’, ‘Often make phone calls with friends or relatives’, ‘Often visit friends or relatives’, and ‘Family or friends are willing to help me to solve problems’. The range of possible scores was 0–20. Better family/friends social support was indicated by a higher score. An ICC of 0.90 and a Cronbach’s alpha score of 0.89 supported the reliability of society dimension of the Chinese version [28]. In this study, the reliability of the social support assessment had a Cronbach’s alpha score of 0.77.

The TUG test is used to determine the basic mobility skill of patients with OA of the knee [29]. The TUG test comprises determining the time taken to get up from a standard chair, walk 3 m, turnaround, walk back, and sit down again [30]. Poorer mobility was indicated by a longer TUG test time. Participants were allowed one practice trial before a timed TUG test. The TUG test was proven to have an excellent test-retest reliability and discriminant validity in community-dwelling older people [31]. The ICC of the TUG test was 0.97 in this study.

The stair-climb test (SCT) assesses the lower extremity strength of elderly people. The SCT comprised climbing five stairs to a platform, turning around, and then climbing down [32]. The whole test process was timed until the participant walked down the last step. Longer times indicated weaker muscle strength and poorer physical coordination. Participants were allowed one practice trial before a timed performance. The test-retest reliability and concurrent validity of SCT were determined as excellent in a study of knee OA [33]. In the present study, as assessed using the ICC, the test–retest reliability had a score of 0.96.

### Analysis of the data

SPSS software version 23 (IBM Corp., Armonk, NY, USA) was used for all data analyses. Variables such as demographics, depression, WOMAC, social support, TUG, and SCT were assessed using descriptive statistics, i.e., the mean, standard deviation (SD), frequencies, and percentages. Chi-square test and Wilcoxon test were used for analysis differences between patients with and without depression of different variables. Among the study variables, correlations were determined using Spearman’s correlation coefficient. The graphical mapping of a correlation matrix was drew with the R Programming Language. Predictors of depression were assessed using stepwise multiple regression analysis. All statistical tests were two-tailed and statistical significance was accepted at *P* ≤ 0.05.

## Results

### Subject characteristics and health conditions

Initially, this study enrolled 219 patients; however, outcome variables were missing for five patients, who were thus excluded. The remaining 214 elderly people with OA of the knee were included in the final analysis. Most of the participants were female (81.8%), married (82.7%), and of Han ethnicity (95.8%). Their average age was 69.2 ± 7.63 (standard deviation) years old, their BMI was 25.2 ± 3.85, and their disease duration was 5.9 ± 7.72 years. A high proportion of the participants (84.1%) had at least one chronic condition, such as osteoporosis, diabetes, heart disease, or hypertension (Table 1).

**Table 1:** Demographic characteristics, Disease, Social support, Timed up and go test, and Stair-climb test of the sample (*n* = 214)

Variables	Mean ± SD or n (%)	Variables	Mean ± SD or n (%)
Age (years)	69.19 ± 7.63	Medical expense	
Sex		Public expense	41 (19.1)
Male	39 (18.2)	Medical insurance	163 (76.2)
Female	175 (81.8)	At their own expense	10 (4.7)
BMI	25.19 ± 3.85	Disease duration (years)	5.86 ± 7.72
Ethnic group		Number of diseased knees	
Han	205 (95.8)	1	94 (43.9)
Others	9 (4.2)	2	120 (56.1)
Marital status		Frequency of fall in past year	
Married	177 (82.7)	0	152 (71.0)
Divorced/widowed	37 (17.3)	1	46 (21.5)
Residence		2 or more	16 (7.5)
Urban	126 (58.9)	Using walking aids	
Rural	88 (41.1)	No	206 (96.3)
Education		Yes	8 (3.7)
Primary school and below	32 (15.0)	Number of comorbidities	
Junior high school	68 (31.8)	0	34 (15.9)
Senior high school or equivalent	51 (23.8)	1	100 (46.7)
College and above	63 (29.4)	2	38 (17.8)
Level of occupational activity before retirement		3 or more	42 (19.6)
Light physical	97 (45.3)	Take pain relievers	
Hard physical	117 (54.7)	No	136 (63.6)
Monthly Income (yuan)		Yes	78 (36.4)
Lower than 2000	59 (27.6)	Pain (scores)	6.77 ± 3.83
2000–3000	55 (25.7)	Stiffness (scores)	2.15 ± 1.88
3000–4000	47 (22.0)	Physical function (scores)	20.98 ± 13.03
4000–5000	23 (10.7)	Social support (scores)	16.79 ± 2.95
Higher than 5000	30 (14.0)	TUG (seconds)	13.06 ± 3.41
		SCT (seconds)	10.54 ± 3.39

*BMI* body mass index, *TUG* timed up and go test, *SCT* stair-climb test

The WOMAC-determined mean level of knee pain was 6.77 ± 3.83 (range 0–20). Their stiffness levels were 0 to 7 (mean = 2.15 ± 1.88), their physical function scores were 0 to 58 (mean = 20.98 ± 13.03). Their level of social support was 16.79 ± 2.95 (range 6–20). Their mean TUG time was 13.06 ± 3.41 s, and their mean SCT time was 10.54 ± 3.39 s (Table 1).

### Depression

The mean total score of the GDS was 4.43 ± 2.89 (range 0–13). Using a score of 5 as the cutoff point, the prevalence of depression elderly community-dwelling patents with OA of the knee was 43.9% (38.5% (men) and 45.1% (women) (*p* > 0.05). Among these 94 depressed patients, 85 (39.7%) had mild depression and 9 (4.2%) had moderate/severe depression. The three items with the highest degree of depression were “Have you dropped many of your activities and interests?” (*n* = 121), “Do you feel you have more problems with memory than most?” (*n* = 113), and “Do you prefer to stay at home, rather than going out and doing new things?” (*n* = 95). The three items with the lowest degree of depression were “Do you feel pretty worthless the way you are now?” (*n* = 20), “Do you often get bored?” (*n* = 34), and “Do you feel full of energy?” (*n* = 38) (Table 2).

**Table 2:** Geriatric Depression Scale (GDS) scores of the sample (*n* = 214)

Items	Yes, n (%)	No, n (%)
2. Have you dropped many of your activities and interests?	121 (56.5)	93 (43.5)
10. Do you feel you have more problems with memory than most?	113 (52.8)	101 (47.2)
9. Do you prefer to stay at home, rather than going out and doing new things?	95 (44.4)	119 (55.6)
^a^11. Do you think it is wonderful to be alive now?	83 (38.8)	131 (61.2)
^a^5. Are you in good spirits most of the time?	80 (37.4)	134 (62.6)
^a^7. Do you feel happy most of the time?	65 (30.4)	149 (69.6)
^a^1. Are you basically satisfied with your life?	60 (28.0)	154 (72.0)
6. Are you afraid that something bad is going to happen to you?	60 (28.0)	154 (72.0)
14. Do you feel that your situation is hopeless?	54 (25.2)	160 (74.8)
8. Do you often feel helpless?	47 (22.0)	167 (78.0)
15. Do you think that most people are better off than you are?	40 (18.7)	174 (81.3)
3. Do you feel that your life is empty?	39 (18.2)	175 (81.8)
*13. Do you feel full of energy?	38 (17.8)	176 (82.2)
4. Do you often get bored?	34 (15.9)	180 (84.1)
12. Do you feel pretty worthless the way you are now?	20 (9.3)	194 (90.7)

^a^Reverse scoring

### Associations between depression and other variables

**Fig. 1:**
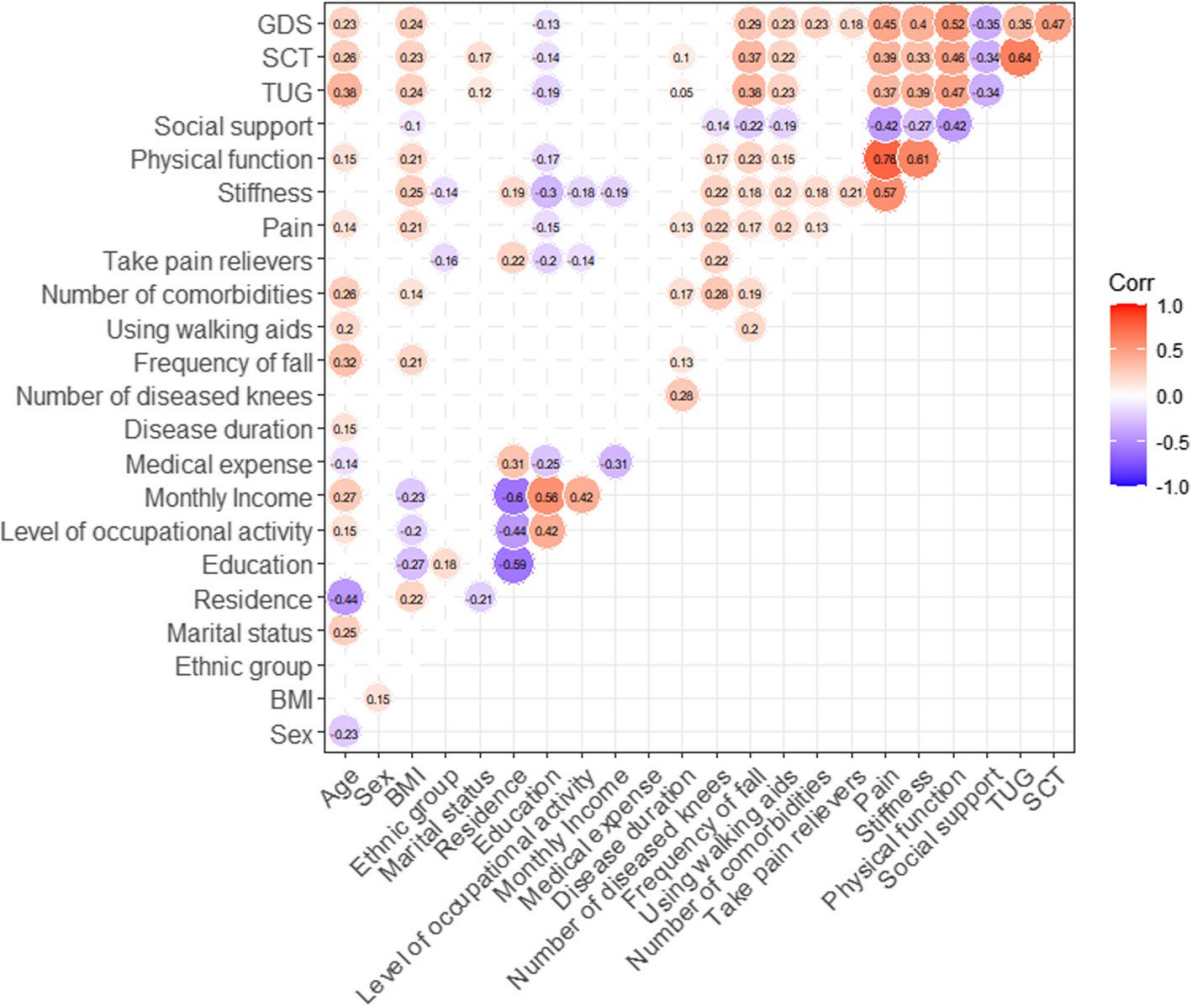
The graphical mapping of the correlation matrix constructed by correlating all influence factors with GDS scores (*P* ≤ 0.05)

There were significant differences between patients with and without depression of different age, BMI, frequency of fall in past year, using walking aids, number of comorbidities, take pain relievers, pain, stiffness, physical function, social support, TUG and SCT (*P* ≤ 0.05) (Table 3). The results of Spearman’s correlation coefficient were similar (Fig. 1). Figure 1 was the graphical mapping of the correlation matrix constructed by correlating all influence factors with GDS scores under the condition of *P* ≤ 0.05. Since all correlations between − 0.3 and 0.3 were to be judged as negligible, the variables of the absolute value of *r* ≥ 0.3 and *P* ≤ 0.05 related to GDS scores from low to high were social support, TUG, stiffness, pain, SCT and physical function. The GDS scores correlated positively with pain (*r* = 0.45, *P* < 0.001), stiffness (*r* = 0.40, *P* < 0.001), physical function (*r* = 0.52, *P* < 0.001), TUG (*r* = 0.35, *P* < 0.001), and SCT (*r* = 0.47, *P* < 0.001) and negatively with social support (*r* = − 0.35, *P* < 0.001). These results indicated that more severe depression correlated with worse pain, stiffness, more serious physical dysfunction, lower mobility, weaker muscle strength and poorer social support.

**Table 3:** Demographic characteristics, Disease, Social support, Timed up and go test, and Stair-climb test in patients with and without depression (*n* = 214)

Variables	With Depression (*n* = 94) Mean ± SD or n (%)	Without Depression (*n* = 120) Mean ± SD or n (%)	*p*-value
Age (years)	71.0 ± 8.36	67.8 ± 6.71	0.002^a^
Sex			0.561
Male	15 (16.0%)	24 (20.0%)	
Female	79 (84.0%)	96 (80.0%)	
BMI	26.2 ± 3.92	24.4 ± 3.63	0.001^a^
Ethnic group			0.734
Han	91 (96.8%)	114 (95.0%)	
Others	3 (3.19%)	6 (5.00%)	
Marital status			0.650
Married	76 (80.9%)	101 (84.2%)	
Divorced/widowed	18 (19.1%)	19 (15.8%)	
Residence			0.747
Urban	57 (60.6%)	69 (57.5%)	
Rural	37 (39.4%)	51 (42.5%)	
Education			0.192
Primary school and below	15 (16.0%)	17 (14.2%)	
Junior high school	31 (33.0%)	37 (30.8%)	
Senior high school or equivalent	27 (28.7%)	24 (20.0%)	
College and above	21 (22.3%)	42 (35.0%)	
Level of occupational activity before retirement			1.000
Light physical	43 (45.7%)	54 (45.0%)	
Hard physical	51 (54.3%)	66 (55.0%)	
Monthly Income (yuan)			0.340
Lower than 2000	25 (26.6%)	34 (28.3%)	
2000–3000	26 (27.7%)	29 (24.2%)	
3000–4000	25 (26.6%)	22 (18.3%)	
4000–5000	9 (9.57%)	14 (11.7%)	
Higher than 5000	9 (9.57%)	21 (17.5%)	
Medical expense			0.062
Public expense	16 (17.0%)	25 (20.8%)	
Medical insurance	70 (74.5%)	93 (77.5%)	
At their own expense	8 (8.51%)	2 (1.67%)	
Disease duration (years)	6.40 ± 8.42	5.43 ± 7.14	0.370
Number of diseased knees			0.060
1	34 (36.2%)	60 (50.0%)	
2	60 (63.8%)	60 (50.0%)	
Frequency of fall in past year			0.001^a^
0	54 (57.4%)	98 (81.7%)	
1	30 (31.9%)	16 (13.3%)	
2 or more	10 (10.6%)	6 (5.00%)	
Using walking aids			0.023^a^
No	87 (92.6%)	119 (99.2%)	
Yes	7 (7.45%)	1 (0.83%)	
Number of comorbidities			0.001^a^
0	5 (5.32%)	29 (24.2%)	
1	48 (51.1%)	52 (43.3%)	
2	17 (18.1%)	21 (17.5%)	
3 or more	24 (25.5%)	18 (15.0%)	
Take pain relievers			0.038^a^
No	52 (55.3%)	84 (70.0%)	
Yes	42 (44.7%)	36 (30.0%)	
Pain (scores)	8.51 ± 3.74	5.40 ± 3.33	< 0.001^a^
Stiffness (scores)	2.93 ± 1.93	1.54 ± 1.60	< 0.001^a^
Physical function (scores)	27.50 ± 12.60	15.90 ± 11.00	< 0.001^a^
Social support (scores)	16.00 ± 2.91	17.40 ± 2.82	< 0.001^a^
TUG (seconds)	14.10 ± 3.73	12.20 ± 2.89	< 0.001^a^
SCT (seconds)	12.00 ± 4.13	9.36 ± 2.01	< 0.001^a^

^a^ represents statistically significant result. BMI, body mass index; *TUG* timed up and go test, *SCT* stair-climb test

### Factors predicting depression

Multiple regression analysis of GDS scores was carried out to assess the predictive abilities of demographic characteristic, scale variables (WOMAC, AIMS2), and test variables (TUG and SCT), on depression. We calculated relevant variables of the absolute value of r ≥ 0.3 and *P* ≤ 0.05. The analysis results showed: 1) physical function and social support variables were entered in step 1; and 2) SCT variables were entered in step 2.

In model 1, 26.1% of the variance in depression was accounted for by the physical function. In model 2, lack of social support increased the variance accounted for by 2.1%. In model 3, the results of the SCT test explained 8.6% of the variance in depression. The full model accounted for 36.8% of the variance in GDS scores (Table 4).

**Table 4:** Multiple regression analysis of depression in elderly patients with osteoarthritis of the knee (*n* = 214)

	Model 1		Model 2		Model 3	
Step predictors	β	t	β	t	β	t
1 Scale variables						
Physical function	0.11	8.74**	0.10	7.13**	0.07	5.00**
Social support			−0.16	−2.64*	−0.09	−1.56
2 Test variables						
SCT					0.29	5.47**
Adjusted R [2]	0.261		0.282		0.368	
Adjusted R [2] change	0.261		0.021		0.086	

*p < 0.01; **p < 0.001. *SCT* stair-climb test

## Discussion

This study investigated the prevalence of depression and the relationships between demographic, disease, social support, mobility, lower extremity strength and depression in elderly patients with knee OA in Chinese communities. Our results showed the prevalence of depression was 43.9%, which appears to be twice compared to the prevalence of 19.9% of depressive symptoms among people with osteoarthritis in a systematic review representing 15,855 individuals [1]. In China, improper pain management and neglect of knee function rehabilitation, inconvenient access to psychotherapy often lead to an unsatisfactory treatment of knee OA [34, 35], which might cause the higher presence of depression in this study. And this high incidence rate of depression in Chinese knee OA elderly is similar to that of Japan, probably because China and Japan are both highly aging societies, other than geographical proximity in eastern Asia, share certain similarities in genetic origins, culture and lifestyles [36]. Similar to our findings, a cross-sectional study by one Japanese team reported a prevalence of 45.3% of depression in older patients with knee OA in Japan, and indicated that increased knee pain and functional limitation might be associated with depression [14].

The current study found that our patients were more likely to be depressed because of dropped many of activities and interests, have problems with memory, and prefer to stay at home, rather than going out and doing new things. The participants’ age and disease might explain this result partially. Aging memory and subsequent depression were seen more often in older people, and high levels of inflammatory disease might interact with depression to contribute to memory difficulties [37]. In addition, activity-induced knee pain and low self-reported physical function, such as decreased lower limb strength, mobility, and balance, might stop the patients going out for activities and doing new things [38]. Meanwhile, dropped activities and new things may reduce the effective social communication of the elderly, and deepen the elderly’s sense of loneliness and uselessness, which may leading to depression [39].

Correlational analyses suggested that depression among elderly patients with OA of the knee was correlated with pain, stiffness, physical function, social support, mobility, and lower extremity strength (TUG and SCT). These results were similar to those reported in previous studies. Chronic pain and depression are highly prevalent in elderly populations worldwide, with 13% of them estimated as suffering from both conditions simultaneously [40]. A post-hoc study reported that co-existing multi-site pain was associated with the presence of depression in patients with symptomatic knee OA [41]. In our current study, we reported similar result. Activity restriction, fear of movement, poorer sleep quality, and negative attitudes caused by knee pain and stiffness of patients with OA of the knee might exacerbate their depressive symptoms [42]. One study showed in community-dwelling older people, independent predictors of depressive symptoms comprised frailty and poor self-rated general health [43]. For patients with OA of the knee, loss of movement and function might limit their day-to-day activities, such as stair climbing, walking, and doing household chores. These conditions might diminish the patients’ quality of life and might be associated with depression and disturbed sleep, which additionally contribute to disability [44, 45]. Previous research showed a significant negative association between social support and the prevalence of depression among the elderly [46]. Our research was similar to it, the fear of movement may prevent participation in exercise and social events, which could lead to further physical and social isolation, and then form poor psychological outlook. The TUG and SCT scores indicated that mobility skill and lower extremity strength were also important correlative factors of depression. This may be explained by that the initiation, progression, and severity of knee OA have been associated with alterations in joint biomechanics and decreased muscular strength [47]. Loss of mobility skill and leg muscular strength was associated with increased disability, causing patients with OA of the knee anxiety, depression and worry about prognosis.

In this study, 36.8% of the total variance in depression could be explained by physical function, social support, and SCT. Higher levels of depression in community-dwelling elderly patients with OA of the knee could be explained by having worse physical function, a low level of social support, and weak lower extremity strength. In particular, physical function made substantial contributions to explaining the prevalence of depression and could represent important predictors. The cartilage of older adults may no longer have the ability to adapt to load bearing because of degenerative changes [48]. WOMAC physical function subscales of self-evaluation assess the difficulty of daily activities for patients such as standing, walking, going up and down stairs, bending over, wearing and taking off socks, doing housework, shopping, getting on and off the bus, and so on [25]. However, these situations were serious issues for persons with knee OA because activities such as squatting, stair climbing, and kneeling may load the tibial-femoral cartilage surfaces in areas that cannot tolerate the load [49]. Perceived instability, lower limb muscle weakness and functional limitations are common downstream effects of this degenerative process [50]. Physical dependency, self-efficacy declines, quality of life deteriorates, social isolation and depression may ensue over time. Previous study proved an exercise program consisting of lower extremity strengthening, stretching, range of motion, balance and agility, and aerobic exercises had beneficial effects on depression in subjects with OA of the knee [51]. More research are needed to understand the association between depression and physical function, social support, and lower extremity strength of elderly with OA of the knee.

### Limitations

This study had some limitations. First, this study only recruited 214 elderly participants from three communities in Beijing, who may not be representative of all geriatric patients with OA of the knee in China. Second, patients with severe OA of the knee could not finish the tests and were thus not interviewed. These patients may suffer from more moderate to severe depression than our participants, especially among older women. Third, patients with musculoskeletal disorders other than knee OA were not excluded. If patients suffered from other chronic musculoskeletal disorders, this might also contribute to the status of depression among this geriatric population. Last, no adjustment was used in the multiple testing performed.

## Conclusion

Our findings suggested that physical function, social support, and lower extremity strength were predictors of depressive symptoms in community-dwelling elderly people with OA of the knee. Focusing on this elderly group with increasing functional exercise, positive social interaction and support, and lower limb muscle strength training should help in the prevention of depression.

## Supplementary Information

**Additional file 1.**

**Additional file 2.**

## Data Availability

The dataset supporting the conclusions of this article is included within the article (Additional file 1).

## Abbreviations

AIMS2: Arthritis Impact Measurement Scales 2
BMI: Body mass index
GDS: Geriatric Depression Scale
ICC: Intraclass correlation coefficients
OA: Osteoarthritis
SCT: Stair-climb test
SD: Standard deviation
TUG: Timed up and go test
WOMAC: Western Ontario and McMaster Universities Osteoarthritis Index
